# Rapidly Progressive Felty Syndrome After Sudden Discontinuation of Methotrexate: A Case Report and Review of Literature

**DOI:** 10.2147/IMCRJ.S365004

**Published:** 2022-09-02

**Authors:** Suaad Hamsho, Isam Alannouf, Anas A Ashour

**Affiliations:** 1 Faculty of Medicine, Damascus University, Damascus, Syria; 2 Department of Internal Medicine, Hamad Medical Corporation, Doha, Qatar

**Keywords:** felty syndrome, rheumatoid arthritis, DMARDs, portal hypertension, case report

## Abstract

Felty syndrome (FS) is a disorder that develops after a long history of erosive rheumatoid arthritis and presents with splenomegaly and neutropenia. In addition to joint deformities, FS causes a variety of extra-articular manifestations such as vasculitis, skin lesions, and lymphadenopathy. FS is also reported to cause non-cirrhotic portal hypertension which may result in variceal bleeding. FS is usually treated by disease-modifying anti-rheumatic drugs (DMARDs) such as methotrexate. Herein, we report a case of rapidly deteriorating FS and a severe relapsing neutropenia only a few weeks after discontinuation of methotrexate and other available DMARDs. The patient presented with a fever and a multi-drug resistant gangrenous ulcer consistent with ecthyma gangrenosum. The patient was also found to have hepatosplenomegaly and portal hypertension. The case was managed with antibiotics and symptomatic treatments only as DMARDs were either unavailable or not affordable by the patient. However, the patient's condition did not improve. This case highlights that DMARDs are considered an essential part of preventing infections due to FS neutropenia. Patients with FS should continue DMARDs for life to avoid the relapse of their condition.

## Introduction

Felty syndrome (FS) is described as a rare syndrome characterized by the triad of rheumatoid arthritis (RA), neutropenia, and splenomegaly.^1^ FS usually develops after a long course of at least 10 years of RA and accounts for <1% of RA patients.^2^ The prevalence of FS declined significantly in the past decades partially due to the introduction of methotrexate (MTX) for the treatment of RA.^3^ Patients with RA can be diagnosed with FS when splenomegaly is detected by physical examination or imaging studies, leukopenia, neutropenia, or thrombocytopenia are present, and when no other known causes for cytopenia or splenomegaly can be identified.^4^ If left untreated, multiple extra-articular clinical conditions can develop including the rare association with idiopathic noncirrhotic portal hypertension.^5^

FS neutropenia can be treated with disease-modifying anti-rheumatic drugs (DMARDs), with MTX being the most frequently used medication.^2^ MTX has been shown to be effective in treating FS in several case series.^6–8^ Herein, we report a case of rapidly progressive FS shortly after the abrupt discontinuation of MTX and other available DMARDs. The patient presented with multiple manifestations of FS including neutropenic fever with a multi-drug resistant skin infection. In addition, rare manifestations of FS such as hepatomegaly with non-cirrhotic portal hypertension were present.

## Case Presentation

A 67-year-old Syrian female, with a known history of RA, presented to AlAssad University Hospital, Damascus, Syria in August, 2021, with a complaint of high-grade fever of 39° C (102° F) for 2 days. On further questioning, the fever was associated with chills and a painful blackish ulcer on her left thigh that she noticed a few days ago. The patient denied having a cough, upper respiratory tract infection symptoms, headache, abdominal pain, or diarrhea. The patient reported having malaise, fatigue, and night sweats. The patient does not recall her previous weight but noticed that she is losing weight. The patient denied having contact with sick patients and does not have a history of recent travel.

Past medical history included a 20-year history of RA associated with joint deformities. The patient was on MTX for RA but in January 2018, MTX was discontinued as it was not available. She was admitted to the hospital in March 2018 due to fever and malaise. Evaluation showed splenomegaly and pancytopenia all of which were not present in previous investigations. She was evaluated for lymphoma at that time. However, her bone marrow biopsy results and lymph node ultrasound showed no signs of lymphoma. The patient was switched to sulfasalazine which she took until 2019, after which it was substituted with azathioprine when sulfasalazine was no longer available as well. Azathioprine was stopped three weeks prior to her current admission. In addition, the patient was diagnosed with brucellosis and vertebral compression fracture in 2020. Both conditions were treated but her evaluation at that time revealed splenomegaly and pancytopenia which persisted after treatment.

Past surgical history was not significant for any major surgeries. Family history was unremarkable for rheumatologic diseases. The patient is a non-smoker, non-alcoholic and eats a regular diet. She is unemployed and is married with children.

**Figure 1:**
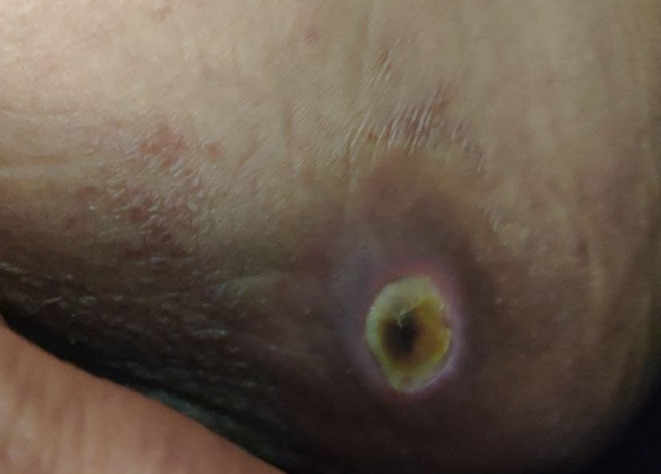
Left upper medial thigh gangrenous ulcer with a central black eschar surrounded by an erythematous halo.

**Figure 2:**
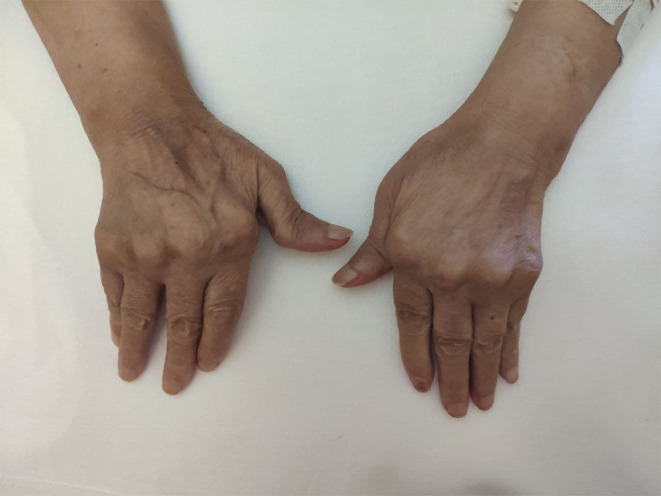
Joint deformities of the hands.

Physical exam revealed a gangrenous ulcer with a central black eschar surrounded by an erythematous halo, located on the medial aspect of her left upper thigh consistent with ecthyma gangrenosum (Figure 1). Rheumatologic examination also revealed deformities in her joints typical of RA, which included z-shaped deformities of both thumbs and ulnar deviation of both hands (Figure 2). It also showed limited flexion and extension of the wrists, limited extension of the elbows, limited abduction, extension, and external rotation of the shoulders, most notably the right shoulder. In addition, synovial thickening of the left wrist and right elbow were present. An abdominal exam showed hepatosplenomegaly. Examination of other systems was unremarkable.

On admission, complete blood count showed white blood cell count of 2.89×10^9^/L with an absolute neutrophil count of 375/mm^3^, hemoglobin of 97 g/L and a platelet count of 110×109/L. The patient had an elevated erythrocyte sedimentation rate of 60 mm/h and C-reactive protein levels of 33 mg/L. Liver transaminases levels were normal. She had a total protein level of 7.3 g/dL and an albumin level of 2.7 g/dL. Bilirubin and lactate dehydrogenase levels were normal. Serology for human immunodeficiency virus (HIV), hepatitis B and hepatitis C antibodies and a purified protein derivative (PPD) skin test produced negative findings. The patient was screened for brucella, due to her past medical history, which was also negative. The antinuclear antibody titer was 1:320, and rheumatoid factor was 113 IU/mL. Levels of complement C3 and C4 were normal. Her urine analysis and culture showed a urinary tract infection (UTI), caused by E. coli. The thigh ulcer was swabbed, and cultures were positive for pseudomonas aeruginosa which turned out to be resistant to all available antibiotics including piperacillin/tazobactam except colistin.

**Figure 3:**
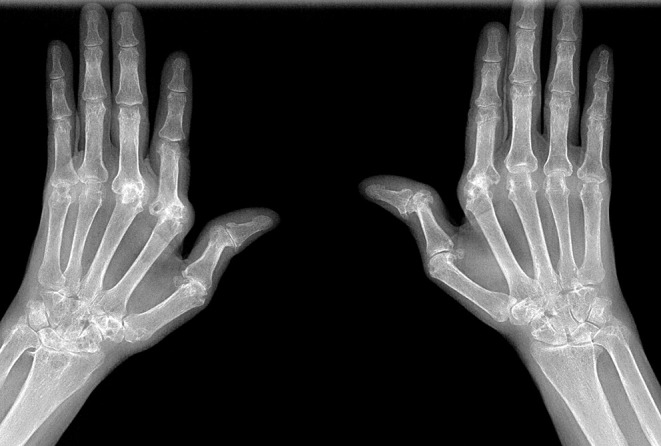
X-ray of the hands showing joint deformities with surrounding osteopenia.

X-rays of the joints of the hands and wrists revealed deformed and eroded ulnar styloid processes resulting in ulnar deviation. The proximal interphalangeal joints and wrists showed severe erosions. The X-ray also revealed osteopenia around the metacarpophalangeal joints (Figure 3). Abdominal ultrasound with Doppler showed hepatomegaly with a midaxillary line length of 16.7 cm with regular margins. There was no intrahepatic biliary tree dilation and hepatic veins were normal. The inferior vena cava showed an irregular endothelial surface with a diameter of 16 mm. The portal vein diameter measured 10 mm with dilation of portal vein tributaries. The spleen measured 14.5 cm, with homogenic echotexture. These findings were consistent with noncirrhotic portal hypertension. A previous abdominal ultrasound done in 2018 was within normal limits except for splenomegaly measuring 14 cm.

Bone marrow aspiration was performed to exclude other hematological disorders. It showed hypercellularity relative to age, maturation arrest with increased metamyelocytes and erythroblasts (<10%), and erythroid series. Megakaryocytes showed acceptable maturation, and plasmacytes were less than 2%. Bone marrow biopsy showed trilineage maturation with mild left shift in granulocyte series and mild focal fibrosis. These findings are consistent with FS but not suggestive of myeloproliferative or lymphoproliferative disease.^2^

To exclude solid organ tumors, a high resolution CT scan of the chest was performed and revealed mild reticular opacities with lower zone predominance, consistent with RA. Abdominal and pelvic MRI revealed an old vertebral compression fracture at the level of L4 with several spinal degenerative changes.

The patient was diagnosed with FS after excluding other possible causes. She was started on empirical piperacillin/tazobactam on admission to treat the gangrenous ulcer on her thigh and the UTI. The patient was offered to start MTX again to control FS neutropenia, but she refused due to the high cost of the medication. In addition, granulocyte colony-stimulating factor (G-CSF) was offered as an alternative option but was also not affordable as well. The patient was started on colistin for the treatment of the skin ulcer. However, the ulcer did not improve, and the patient was discharged home after a few days of hospitalization with symptomatic treatments only.

## Discussion

We report a case of severe relapsing FS that exacerbated a few weeks after the discontinuation of DMARDs. The patient presented to the hospital in 2018 with a full blown FS only 2 months after discontinuation of MTX. The patient presented again in the last admission with multi-drug resistant skin infections 3 weeks after discontinuation of azathioprine. Due to the rarity of this disease, most of the available clinical guidelines are based on case reports and small case series. As a result, the efficacy and safety of many of the available drugs used for RA is not well-studied in patients with FS. Our case illustrates the clear role of DMARDs, particularly MTX, in preventing extra-articular manifestations of FS. One of the most serious manifestations of FS which was seen in this case is the recurrent and resistant bacterial infections secondary to neutropenia. The pathophysiology of FS neutropenia is not yet clear. One of the suggested mechanisms linked it to both decreased granulopoiesis and increased degradation of granulocytes in peripheral blood.^9^ Another hypothesis proposed a role of cellular and humoral immunological mechanisms in the pathogenesis of FS neutropenia.^9^ DMARDs including methotrexate, G-CSF, and glucocorticoids are considered the mainstay of FS neutropenia therapy.^10^ MTX is considered the most effective, and well tolerated option among DMARDs in treating both RA symptoms and neutropenia. Hence, it is considered the first line of therapy in such patients.^8^,^9^ In addition, multiple case reports published recently reported successful treatment of FS with rituximab.^11^ Leflunomide, and sulfasalazine can also be used in the management but should be considered as a second line due to the limited experience.^12^,^13^ Azathioprine is generally not recommended in patients with FS due to the limited data on efficacy. In a study that assessed the effect of multiple medications on FS, azathioprine was effective in only one out of seven patients.^14^ If all medical options fail to produce a significant effect in treating neutropenia, splenectomy could be considered as it can achieve a long-term response in 80% of cases.^10^

Another rare manifestation of FS that was seen in this case is non-cirrhotic portal hypertension. Thorne et al (1982) examined 18 cases with FS by liver biopsy and demonstrated that 12 patients had abnormal liver histology.^5^ However, only 4 of those patients had portal hypertension. Thorne also reported that 3 out of the 4 patients with portal hypertension developed esophageal varices, a serious and life-threatening consequence of portal hypertension. This was also seen in a recently published case that reported bleeding esophageal varices as a complication of FS.^15^ The prevalence of liver manifestations in patients with FS might be much less than the figures reported by Thorne et al (1982) due to the routine use of MTX and other effective treatment modalities that were introduced in the last 3 decades.^5^ However, FS should not be overlooked as a possible cause of elevated liver enzymes or portal hypertension in patients with RA. The mechanism of portal hypertension in FS has not been fully elucidated. Suggested possible mechanisms include increased splenic blood flow, obliterative liver fibrosis of the portal branches, or autoimmune injury.^16^,^17^

The devastating consequences of the war in Syria has severely affected the healthcare system in multiple ways.^18^ The availability of healthcare facilities and access to medical supplies and drugs was limited.^18^ Compared to other fields of medicine, oncologic and rheumatologic medications, especially biologics, were affected the most due to their high cost and lack of local alternatives.^19^ In addition, clinical guidelines that are published from developed countries often fail to discuss alternative options for less resourced countries. Recently, efforts by the rheumatology community have been made to address management of some rheumatologic diseases in less resourced countries.^20^ In our reported case, lack of affordable and available therapeutic options has led to such an unfavorable clinical outcome.

## Conclusion

Patients suspected of having Felty syndrome should be prescribed DMARDs soon after confirming the diagnosis, and patients' adherence to these medications should be highly recommended. Clinical guidelines should take into consideration possible alternative therapies for less resourced countries.
